# Polygenic Risk Scores for Incident Dementia in the Multi‐Ethnic Study of Atherosclerosis

**DOI:** 10.1002/gepi.70046

**Published:** 2026-06-23

**Authors:** Diane Xue, Elizabeth E. Blue, Tamar Sofer, Timothy M. Hughes, Jerome I. Rotter, Wendy S. Post, Alison E. Fohner

**Affiliations:** ^1^ Department of Genetics University of Pennsylvania Philadelphia PA USA; ^2^ Division of Medical Genetics, Department of Medicine University of Washington Seattle WA USA; ^3^ Institute for Public Health Genetics University of Washington Seattle WA USA; ^4^ Brotman Baty Institute Seattle WA USA; ^5^ CardioVascular Institute, Beth Israel Deaconess Medical Center, Harvard Medical School Boston MA USA; ^6^ Department of Biostatistics Harvard T.H. Chan School of Public Health Boston MA USA; ^7^ Department of Internal Medicine, Section on Gerontology and Geriatric Medicine Wake Forest University School of Medicine Winston‐Salem NC USA; ^8^ Department of Pediatrics, The Lundquist Institute for Biomedical Innovation at Harbor‐UCLA Medical Center Institute for Translational Genomics and Population Sciences Torrance CA USA; ^9^ Division of Cardiology, Department of Medicine Johns Hopkins University Baltimore MD USA; ^10^ Department of Epidemiology University of Washington Seattle WA USA

**Keywords:** Alzheimer's disease, dementia, diversity, multi‐ancestry, polygenic risk score

## Abstract

Over 75 Alzheimer's disease (AD) and dementia‐associated variants have been identified through genome‐wide association studies, but the utility of polygenic risk scores (PRS) for predicting AD and dementia in diverse and admixed populations remains unclear. We compared how PRS approaches differing in *p*‐value thresholds, variant weights, and source ancestry perform in predicting dementia in 6338 African American, Chinese, Hispanic, and White individuals from the Multi‐Ethnic Study of Atherosclerosis. We tested clumping and thresholding (C+T) methods with varying parameters against Bayesian approaches (PRS‐CS, PRS‐CSx). We compared the ability of each method to predict incident dementia in all participants and in groups stratified by self‐reported race/ethnicity. We additionally analyzed performance across groups stratified by estimated proportion of non‐Finnish European (NFE)‐like ancestry. Including more variants does not improve performance. We found comparable associations between dementia and PRS when comparing a C+T method with only 15 SNPs and PRS derived from Bayesian models that include > 800,000 SNPs (HR_5e‐08_ = 1.18, 95% CI: 1.08–1.28; HR_CSx_ = 1.17, 95% CI: 1.07−1.27). The *p* < 5e‐08 C+T method was more strongly associated with incident dementia in populations genetically dissimilar from the source data (HR_lowNFE___5e‐08_ = 1.27, 95% CI: 1.08−1.50; HR_lowNFE___CSx_ = 1.12, 95% CI: 0.94−1.33). More selective PRS models using genome‐wide significant SNPs may be preferable for dementia prediction in diverse populations.

## 1 Introduction

Dementia is a growing global health challenge, projected to affect over 150 million people worldwide by 2050 (Nichols et al. 2022). Populations are aging around the world, and with nearly one‐third of adults over 65 dying with Alzheimer's disease (AD) or other dementias, there is an urgent need for effective predictive tools that can aid in risk stratification and lead to more precise treatment and prevention (“2024 Alzheimer's Disease Facts and Figures” 2024). AD is the most common cause of dementia and is strongly influenced by genetic variation. Less than one percent of AD cases have early‐onset autosomal dominant forms of disease caused by rare coding changes in *APP, PSEN1*, or *PSEN2* (Campion et al. 1999). The vast majority of cases have a far more complex etiology. The apolipoprotein E *(APOE) ɛ4* allele is the strongest genetic risk factor for late‐onset AD, but less than half of AD patients carry an *ɛ4* allele, and effects are attenuated for alleles on African haplotypic backgrounds (Bertram et al. 2008; Blue et al. 2019; Pericak‐Vance et al. 1991; Rajabli et al. 2018). Aside from *APOE*, dozens of loci have been found to be significantly associated with AD through genome‐wide association studies (GWAS) (Andrews et al. 2023). While these loci typically have small effect sizes, they are more common in the population, and their joint effects can place individuals at elevated genetic risk for disease.

Polygenic risk scores (PRS) based on the effects of common genetic variants have been shown to predict AD (de Rojas et al. 2021; Lambert et al. 2019; Leonenko et al. 2021). However, there is no clear consensus on the best approach for constructing PRS for AD or dementia, particularly in diverse and admixed populations. The most traditional approach for constructing PRS is clumping and thresholding (C+T), which involves initially considering all SNPs tested in a GWAS and then filtering them based on *p*‐value threshold and linkage disequilibrium (LD) (Choi et al. 2020). Some previous studies have found that less stringent *p*‐value thresholds, which allow for the inclusion of a larger number of SNPs in the PRS, lead to better predictive performance of AD. Escott‐Price et al. (2015) reported that the PRS with a *p*‐value threshold *<* 0.5 was most strongly associated with AD(Escott‐Price et al. 2015). Another study suggests that a threshold of *p <* 0.10 had optimal performance (Leonenko et al. 2019). In contrast, Zhang and colleagues found that restricting PRS to SNPs that are significantly (*p <* 1e‐08) or suggestively (*p <* 3e‐04) associated with AD in GWAS had better performance, implying PRS constructed using fewer than 100 SNPs can achieve superior prediction (Zhang et al. 2020). Another study found that the optimal *p*‐value differed depending on the target population, ranging from *p <* 0.1 to *p <* 5e‐08 (Bellou et al. 2025). Notably, the comparisons discussed thus far are limited to populations with European ancestry.

Previous studies of other complex traits have shown that PRS performance deteriorates as the genetic distance between the target and GWAS training populations increases (Ding et al. 2023; Martin et al. 2019; Privé et al. 2018). Most of the risk loci discovered to be significantly associated with AD have been found in large GWAS studies of self‐reported non‐Hispanic White individuals who cluster with 1000 Genome (1KG) European references (Bellenguez et al. 2022; Kunkle et al. 2020). Few signals have been replicated in populations with different genetic ancestral backgrounds, including *APOE, ABCA7, TREM2, SORL1*, and *CLU* (Reitz et al. 2023). GWAS of non‐European ancestry populations remain underpowered to discover risk loci with low to moderate effects, which comprise most of the AD risk loci identified thus far in large European ancestry GWAS (Xue et al. 2024). Previous studies have examined the performance of PRS for AD across populations but have limited their comparisons to C+T methods (Jung et al. 2022; Marden et al. 2014; Osterman et al. 2024; Sariya et al. 2021). We hypothesize that PRS performance across populations can be improved by using methods that retain SNPs with potential population‐specific effects that would typically be excluded by pre‐selected *p*‐value and LD thresholds.

In this study, we assess the performance of various PRS methodologies in predicting late‐onset dementia in a diverse cohort. The Multi‐Ethnic Study of Atherosclerosis (MESA), a longitudinal cohort study, includes participants who self‐identify as Black/African American, Chinese, Hispanic, or White. Based on GWAS of clinically ascertained AD, we compared the performance of traditional C+T methods at a range of *p*‐value thresholds against Bayesian approaches (PRS‐CS, PRS‐CSx) using summary statistics from GWAS with differing ancestral backgrounds.

## 2 Methods

### 2.1 Study Population

MESA has been previously described in detail (Bild 2002). Briefly, MESA is a prospective cohort study originally designed to study cardiovascular disease. Between 2000 and 2002, MESA recruited 6814 Black/African American, Chinese, Hispanic/Latino, and White participants aged 45−84 from six sites in the United States: Baltimore, Maryland; Chicago, Illinois; Forsyth County, North Carolina; Los Angeles County, California; Northern Manhattan and the Bronx, New York; and Saint Paul, Minnesota. All participants were free from clinical cardiovascular disease and dementia at baseline. Because MESA enrolled dementia‐free adults aged 45–84 years at baseline, we examined *APOE* genotype frequencies across baseline age strata to assess whether ε4 carrier frequency decreased with older recruitment age. All participants provided written informed consent at baseline and all subsequent exams. Institutional Review Board approval was received from each of the six sites. MESA participants with imputed genotypes and information on dementia status were included in this study.

### 2.2 Inferring Global Ancestry Proportions

We estimated global ancestry proportions for all genotyped MESA participants. Global ancestry proportions are based on local ancestry estimates from RFMix2 using reference population data from the 1KG and Human Genome Diversity Project available through gnomADv3.1 as references (Auton et al. 2015; Karczewski et al. 2020; Maples et al. 2013). Samples were randomly selected from the following superpopulation groups to construct balanced sample maps: American (AMR), African (AFR), East Asian (EAS), and Non‐Finnish European (NFE) (Auton et al. 2015). The genetic map with coordinates from the human reference genome GRCh38 was downloaded from the Eagle v2.4.1 package (http://data.broadinstitute.org/alkesgroup/Eagle/downloads/). Using the global ancestry proportions, participants were assigned to one of three groups based on NFE tertiles, with the low NFE‐like group representing those with the greatest genetic distance from the GWAS training sample.

### 2.3 Dementia Outcome

#### 2.3.1 Incident Possible Dementia in Time‐to‐Event Models

Participants were followed up via telephone interview every 9 to 12 months through 2018 for updates on hospital admissions or deaths. Dementia was identified based on a set of ICD codes at either hospitalization or death and the time of the event was determined based on the first occurrence. The candidate dementia cases were identified using the following diagnosis codes: ICD‐9: 290, 294, 331.0, 331.1, 331.2, 331.82, 331.83, 331.9, 438.0, and 780.93; ICD‐10: F00, F01, F03, F04, G30, G31 (excluding G31.2), I69.91, and R41. The ICD code‐based identification was validated against medical record text that indicates a significant decline in cognitive function compared with a previous level (Fujiyoshi et al. 2017).

#### 2.3.2 Adjudicated Cognitive Impairment in Case‐Control Models

A subset of MESA participants enrolled in the MESA MIND ancillary study (2019‐2024), where they completed detailed cognitive testing using the National Alzheimer's Coordinating Center Uniform Data Set Neuropsychological Battery version 3. A consensus adjudication committee reviewed the cognitive scores, clinical data, and physical examination to categorize participants into the following groups: no impairment, mild cognitive impairment, probable dementia, and cannot classify. In our logistic regression analysis, cases were classified as those who were determined to have probable dementia from the cognitive adjudication or possible dementia based on previously described ICD codes (Supporting Table 1). In case of discrepancies, the cognitive adjudication was used.

### 2.4 Genotyping

SNPs for all MESA participants were genotyped using the Affymetrix 6.0 SNP array. Variant‐level filtering excluded SNPs with call rate below 0.95, Hardy–Weinberg equilibrium *p*‐values less than 1e‐06, monomorphic SNPs, variants missing in more than 5% of samples within any self‐identified racial/ ethnic group, and those with a minor‐allele frequency under 0.01. Individual‐level filtering removed samples with call rate below 0.95, those showing unresolved discrepancies between reported and genetically determined sex, and unresolved duplicates. SNPs were imputed using IMPUTE version 2.2.2 and 1KG cosmopolitan phase 3 version 5 reference haplotypes. Relatedness was inferred using KING, and an unrelated subset of individuals was selected by choosing one individual at random from each first‐degree related group (Manichaikul et al. 2010).

#### 2.4.1 Calculating Polygenic Risk Scores

We compared six PRS models that excluded the *APOE* region (GRCh38 chr19: 44408822 ‐ 45408822). The C+T methods were used to estimate PRS using the following *p*‐value thresholds: 0.01, 1e‐05, and 5e‐08. SNPs were filtered based on LD *< r* ^2^ = 0.01. C+T PRS were calculated using PLINK v1.90 (Chang et al. 2015). After filtering based on *p*‐value and LD, PRS were calculated based on the dosage of the SNP effect allele multiplied by the effect sizes. The SNPs and effect sizes for the C+T models were derived from the 2019 International Genomics of Alzheimer's Project (IGAP) genetic meta‐analysis of clinically diagnosed late‐onset Alzheimer's disease among those of European descent, which includes 21,982 cases and 41,944 controls across 46 studies (Kunkle et al. 2020). Summary statistics were obtained from the National Institute on Aging Genetics of Alzheimer's Disease Data Storage Site (NIAGADS).

In addition to the C+T models with varying *p*‐value stringency, two Bayesian models were compared: PRS‐CS and PRS‐CSx (Ge et al. 2025; Ruan et al. 2022). Both PRS‐CS and PRS‐CSx use a continuous shrinkage model that accounts for LD by tuning or shrinking the effect sizes. We did not use a separate validation set to tune parameters, instead using the ‐auto option for both PRS‐CS and PRS‐CSx. Two GWAS summary statistics were used for the PRS‐CS models: the IGAP study and a cross‐population GWAS of 15,579 cases and 17,690 controls that included self‐reported Whites, African Americans, Japanese, and Israeli‐Arabs (NG00056) (Jun et al. 2022). The 1KG EUR samples were used for LD reference. PRS‐CSx allows for multiple summary statistics with differing ancestral backgrounds to be used concurrently. In addition to the European ancestry IGAP study, we also included summary statistics from the African Genome Resources Panel GWAS of 2748 cases and 5222 controls in the same model (NG00100) (Kunkle et al. 2020). The 1KG EUR and AFR samples were used as LD reference panels for PRS‐CSx.

To calibrate the PRS for population structure, we used a previously described procedure to calculate residualized scores based on the principal components (Hao et al. 2022). We fit the raw PRS as a function of the first three principal components in non‐affected individuals. We used the linear model to calculate a predicted PRS for all individuals. We then computed the residualized, population‐structure adjusted PRS by calculating the difference between the raw and predicted PRS. The residualized score was then standardized based on the mean and standard deviation.

### 2.5 Statistical Analysis

Cox proportional hazards models were used to examine the association between the PRS scores and incident dementia, with age as the time axis. Each participant was considered at risk from their age at entry until age at either first occurrence or censoring. We additionally conducted analyses in groups stratified by self‐reported race/ethnicity to examine whether the PRS models perform unequally across groups, which may exacerbate disparities. We also performed analyses stratified by quantiles of NFE‐like ancestry to evaluate whether PRS performance declines as genetic distance from the training GWAS of primarily European ancestry increases. Univariate models were computed separately for each PRS method.

Harrell's concordance (C‐Index) was used to compare predictive performance. Comparisons were conducted in all participants and groups stratified by self‐reported race/ethnicity. Additional comparisons were made across groups in different tertiles of European ancestry to examine if model performance was biased for those with greater proportion of European ancestry.

We computed the added predictive value of each PRS method compared to baseline survival models for age‐specific dementia prediction that included sex and *APOE ɛ4* carrier status. We used likelihood ratio tests to test the significance of improved model fit when including the PRS.

### 2.6 Sensitivity Analysis

Time to hospitalization or death due to dementia may not accurately capture dementia symptom onset or diagnosis. In addition to the comparisons of association and predictive performance based on hazard models, we also fit logistic regression models to examine the association with probable dementia based on cognitive adjudication or possible dementia based on ICD code if adjudication was unavailable. For each PRS approach, we fit univariate regression models and multiple regression models that adjusted for age, sex, and *APOE* ε4 carrier status. We calculated the area under the curve (AUC) to assess the predictive performance. To assess whether results were affected by the inclusion of non‐AD dementias, we additionally conducted a sensitivity analysis in the time‐to‐event models restricted to AD‐specific ICD codes only (ICD‐9 331.0; ICD‐10 F00 and G30).

## 3 Results

### 3.1 Study Population

We calculated PRS and inferred global ancestry proportions for all participants in the MESA cohort who consented to genetic analyses as part of the SNP Health Association Resource (SHARe). Of these individuals, 6,338 participants had dementia follow‐up data and were included in this study. At enrollment, the mean age of the participants was 62 years (Table 1, Supplementary Table 2). We observed only a modest decline in ε4 carrier frequency from 28.5% at ages 45–54 years to 25.3% at ages 75–84 years, and in ε4/ε4 frequency from 2.7% to 1.8% (Supplementary Table 3). After a median follow up of 16.8 years, 560 (8.8%) incident all‐cause dementia events were observed. Complete demographic characteristics are provided in Table 1. The African Americans and Hispanic/Latino groups have high amounts of admixture of NFE‐like and AFR‐like ancestry and NFE‐like, AMR‐like, and AFR‐like ancestry, respectively. (Supporting Figure 1). The mean proportion of NFE‐like ancestry in the NFE tertile groups are as follows: low‐NFE = 0.10, intermediate‐NFE = 0.54, high‐NFE = 0.91.

**Table 1:** Demographic characteristics of MESA participants at baseline.

	Censored	Dementia	Overall
	(*N *= 5778)	(*N* = 560)	(*N* = 6338)
Age at baseline (years)			
Mean (SD)	61.3 (9.95)	72.2 (7.71)	62.2 (10.2)
Median [Min, Max]	61.0 [44.0, 84.0]	74.0 [45.0, 84.0]	62.0 [44.0, 84.0]
Sex			
Female	3035 (52.5%)	283 (50.5%)	3318 (52.4%)
Male	2743 (47.5%)	277 (49.5%)	3020 (47.6%)
Race/ethnicity			
Black	1457 (25.2%)	146 (26.1%)	1603 (25.3%)
Chinese	734 (12.7%)	40 (7.1%)	774 (12.2%)
Hispanic/Latino	1327 (23.0%)	115 (20.5%)	1442 (22.8%)
White	2260 (39.1%)	259 (46.3%)	2519 (39.7%)
Level of education			
*<* High school degree	1030 (17.8%)	126 (22.5%)	1156 (18.2%)
High school degree	1021 (17.7%)	137 (24.5%)	1158 (18.3%)
Some college, no bachelor's degree	1639 (28.4%)	141 (25.2%)	1780 (28.1%)
Bachelor's degree or higher	2069 (35.8%)	155 (27.7%)	2224 (35.1%)
Missing	19 (0.3%)	1 (0.2%)	20 (0.3%)
APOE genotype			
*ɛ2/ɛ2*	45 (0.8%)	2 (0.4%)	47 (0.7%)
*ɛ2/ɛ3*	694 (12.0%)	58 (10.4%)	752 (11.9%)
*ɛ2/ɛ4*	147 (2.5%)	14 (2.5%)	161 (2.5%)
*ɛ3/ɛ3*	3471 (60.1%)	296 (52.9%)	3767 (59.4%)
*ɛ3/ɛ4*	1196 (20.7%)	160 (28.6%)	1356 (21.4%)
*ɛ4/ɛ4*	128 (2.2%)	20 (3.6%)	148 (2.3%)
Missing	97 (1.7%)	10 (1.8%)	107 (1.7%)

### 3.2 PRS Distributions

Table 2 outlines the number of SNPs included in each PRS model. The PRS‐CSx model incorporated the largest number of SNPs (968,595) that intersect across the 1KG LD reference maps, IGAP European ancestry GWAS summary statistics, African Genome Resources Panel GWAS summary statistics, and MESA genotyped + imputation data. The PRS‐CS models with the European IGAP GWAS and cross‐population GWAS included 862,647 SNPs and 851,128 SNPS, respectively. In contrast, the C+T model with a stringent genome‐wide significant *p*‐value cutoff (*p <* 5e‐08 C+T) included only 15 SNPs after filtering for LD.

**Table 2:** Description of PRS models.

Model	Method	*P*‐value cutoff	# SNPs	GWAS ancestry	GWAS Cases/Controls	GWAS study name [NIAGADS study ID]
**1**	C+T	5e‐08	15	NFE‐like	21,982/41,944	International Genomics of Alzheimer's Project [NG00075, 2019]
**2**		1e‐05	53			
**3**		0.1	9023			
**4**	PRS‐CS	NA	862,647			
**5**			851,128	Multi‐ancestry^a^	15,579/17,690	ADGC multi‐ancestry [NG00056, 2017]
**6**	PRS‐CSx		968,595	NFE‐like	21,982/41,944	[NG00075, 2019]
				AFR‐like	2748/5222	African Genome Resources Panel [NG00100, 2021]

*Note:* This table shows the six PRS models being compared. The models differ in computational method, *p*‐value threshold, number of SNPs included, and/or GWAS training data. The NIAGADS study number is provided for the GWAS summary statistics used. LD reference panels were obtained from 1000 Genomes Project phase 3 samples (EUR and AFR) and used for the PRS‐CS (EUR) and PRS‐CSx (EUR and AFR) models.

a Populations represented in the multi‐ancestry GWAS include European, African American, Japanese, and Israeli‐Arab.

**Figure 1:**
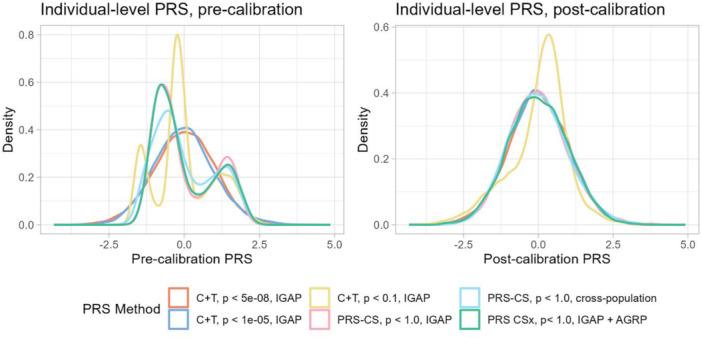
PRS distributions before and after calibration by principal components. Density plots show the distribution of risk scores for each PRS method across all participants. (Left) Distributions of scores that have been mean‐standardized, not adjusted for principal components. (Right) Distributions of scores after standardization and principal component calibration.

For all models, marked differences in PRS distributions were observed across self‐reported racial groups using raw scores, although the differences are less pronounced in the *p <* 5e‐08 C+T model. The variation was attenuated after calibrating for population structure. (Figure 1, Supporting Figure 2).

### 3.3 Association Between PRS and Incident Dementia

**Figure 2:**
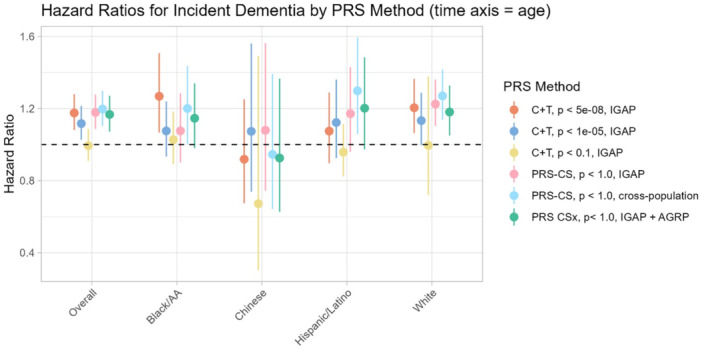
Association between PRS and incident dementia. The forest plots display the hazard ratio and confidence intervals for univariate models with the PRS as exposure and incident dementia outcome. Results are presented for all participants (Overall) and for groups stratified by self‐reported race/ethnicity.

Univariate Cox proportional hazards models were fit to test the association of each PRS model with incident dementia. In the full sample, PRS derived from all models except for the C+T model with *p*‐value threshold *<* 0.1 were associated with incident dementia (Figure 2). The hazard ratios were not significantly different across the associated models (HR_5e‐08_ = 1.18, 95% CI: 1.08−1.28; HR_1e‐05_ = 1.12, 95% CI: 1.02−1.21; HR_CS‐EUR_ = 1.18, 95% CI: 1.09−1.28; HR_CS‐CP_ = 1.20, 95% CI: 1.10−1.30; HR_CSx_ = 1.17, 95% CI: 1.07−1.27; Figure 2).

Among the race/ethnicity‐stratified models, the cross‐population PRS‐CS model was associated with dementia in Hispanic/Latino (HR_HIS_CS‐CP_ = 1.30, 95% CI: 1.06−1.60) and White groups (HR_WHI_CS‐CP_ = 1.27, 95% CI: 1.14−1.42). The *p <* 5e‐08 C+T model was associated with incident dementia among the African American/Black (HR_AA_5e‐08_ = 1.27, 95% CI: 1.07−1.51) and White participants (HR_WHI_5e‐08_ = 1.20, 95% CI: 1.06−1.36). No PRS were significantly associated with dementia in the Chinese participants, likely due to the limited number of observations (40 cases, 734 censored).

Under the assumption that model performance may be more dependent on genetic similarity to the GWAS sample than self‐reported race/ethnicity, we also stratified MESA participants into tertiles of NFE‐like ancestry (Supporting Figure 3). We found that only the most stringent *p <* 5e‐08 C+T was associated with incident dementia in the low and high NFE‐like group (HR_lowNFE_5e‐08_ = 1.27, 95% CI: 1.08−1.50; HR_highNFE_5e‐08_ = 1.27, 95% CI: 1.07−1.50). No PRS was associated with the incident dementia in the intermediate NFE‐like group, regardless of approach.

In a sensitivity analysis restricting cases to those with AD‐specific ICD codes (*N* = 185), hazard ratio estimates were broadly similar to those from the primary all‐cause dementia analysis, with the same general pattern across PRS methods, although confidence intervals were wider because of the smaller number of AD‐specific events (Supporting Figure 4).

As a sensitivity analysis, we fit univariate and multiple logistic regression models to assess the association between the PRS models and dementia. The results from the logistic regression models are similar to those from the Cox proportional hazards models. Among all MESA participants, PRS derived from all models except for the C+T model with *p*‐value threshold *<* 0.1 were associated with probable or possible dementia in the univariate and multiple regression models (Supporting Figure 5). The odds ratios were not significantly different across the five associated models (OR_5e‐08_ = 1.17, 95% CI: 1.08−1.28; OR _1e‐05_ = 1.10, 95% CI: 1.01−1.19; OR _0.01_ = 0.97, 95% CI: 0.89−1.06; OR _CS‐EUR_ = 1.20, 95% CI: 1.11−1.30; OR _CS‐CP_ = 1.20, 95% CI: 1.11−1.30; OR _CSx_ = 1.15, 95% CI: 1.06−1.25).

### 3.4 Assessing Model Performance

**Figure 3:**
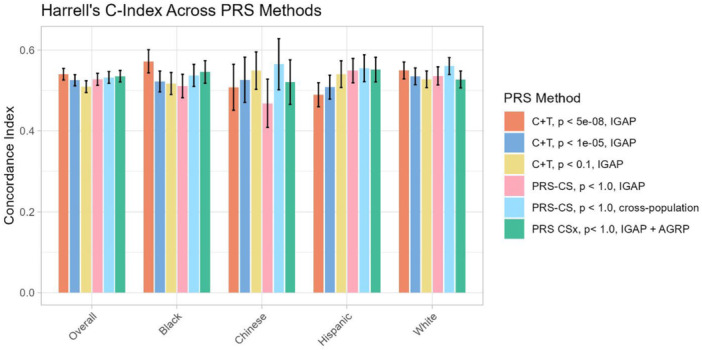
PRS predictive performance measured by Harrell's Concordance Index. The bar plots show a comparison of prediction accuracy as measured by the concordance index or Harrell's C. Error bars represent 95% confidence intervals.

Model performance was evaluated using Harrell's concordance index (C‐index), with the highest value observed for the most stringent C+T model (C_5e‐08_ = 0.54, standard deviation (SD) = 0.01). Comparisons across models revealed that the inclusion of more SNPs in either C+T models or using Bayesian approaches does not improve predictive accuracy (Figure 3). These findings were also supported by comparisons of model AUC (AUC_5e‐08_ = 0.55, SD = 0.01, Supporting Figure 6).

In groups with low proportions of NFE‐like ancestry, the *p <* 5e‐08 C+T model had the highest C‐index (C_lowNFE_5e‐08_ = 0.56, SD = 0.03, Supporting Figure 7). In the group with intermediate NFE‐like proportion, the PRS‐CSx model had the best performance (C_midNFE_csx_ = 0.54, SD = 0.02) while the *p <* 5e‐08 C+T model had the worst performance (C_midNFE_5e‐08_ = 0.49, SD = 0.02). In the high NFE‐like group, PRS‐CS using the cross‐population training GWAS had superior predictive performance, followed by the *p <* 5e‐08 C+T and PRS‐CS with EUR training GWAS models (C_highNFE_CS‐CP_ = 0.59, SD = 0.02; C_highNFE_5e‐08_ = 0.55, SD = 0.02; C_highNFE_CS‐EUR_ = 0.55, SD = 0.02).

Harrell's C‐index values in the AD‐specific ICD sensitivity analysis were similar in overall magnitude to those from the primary analysis, although the ordering of PRS methods differed somewhat across subgroups, likely reflecting greater variability due to the smaller number of AD‐specific events (Supporting Figure 8).

### 3.5 Value Added From Prediction Using PRS

Compared to a baseline model for age‐specific dementia prediction that included sex and *APOE* ε4 carrier status, the addition of PRS derived from *p <* 5e‐08 and *p <* 1e‐05 C+T models and the PRS‐CSx model led to a marginal increase in C‐index. The baseline model had a C‐index of 0.58. In the *p <* 5e‐08 C+T model and PRS‐CSx model, inclusion of the PRS significantly improved model fit (*p*_LRT_5e‐08_ = 0.0001, *p*_LRT_CSx_ = 0.001, Table 3). The remaining PRS models did not improve model fit.

**Table 3:** Value added of PRS.

PRS model	Δ Concordance	*p*‐value_LRT_
C+T, *p < *5e‐08	0.0069	0.0001^a^
C+T, *p < *1e‐05	0.0059	0.012
C+T, *p < *0.01	0.0038	0.855
PRS‐CS, IGAP	0.0012	0.204
PRS‐CS, Cross‐Pop	0.0005	0.128
PRS‐CSx	0.0069	0.001^a^

*Note:* We computed the added value for age‐specific dementia prediction of each PRS method compared to baseline models that included sex and *APOE* ɛ4 carrier status. We used likelihood ratio tests to test the significance of improved model fit when including the PRS. This table shows the change in concordance (C‐statistic) from adding PRS to the model and the *p*‐value corresponding to the likelihood ratio test.

a statistically significant *p*‐values (α = 0.008).

## 4 Discussion

Improved prediction of Alzheimer's disease and dementia is urgently needed for advancing research into novel treatment and prevention strategies. PRS are increasingly being used to assess genetic susceptibility for a wide spectrum of diseases, allowing for earlier identification of individuals at higher risk, but few studies have assessed the association of Alzheimer's disease PRS for predicting dementia in diverse populations. Our study demonstrates that PRS models, even when excluding the *APOE* region, remain significantly associated with incident dementia in a multi‐ancestry sample. We also found that including more SNPs does not improve predictive performance. The C+T *p* < 5e‐08 model and Bayesian approaches PRS‐CS and PRS‐CSx with varying GWAS training datasets had comparable predictive performance and association with dementia. These results are similar to those demonstrated by previous comparisons of PRS approaches for Alzheimer's disease prediction. Most recently, Nicolas *et al*. found that PRS‐CSx did not outperform C+T across diverse populations while Bellou et al. found comparable predictive performance for dementia when comparing C+T methods with PRS‐CS in European ancestry test sets (Bellou et al. 2025; Nicolas et al. 2024). While Bellou et al. found that the optimal *p*‐value thresholds for C+T models varied by target dataset, ranging from 0.1 to 5e‐08, other studies have demonstrated that more stringent *p*‐value thresholds lead to improved C+T model performance(Leonenko et al. 2019; Zhang et al. 2020). In line with these findings, our analyses also showed improved C+T performance when using a genome‐wide significant *p*‐value cutoff. However, even the best performing PRS models in our study have low predictive power (C‐index *<* 0.6) and add only slight improvements to age‐specific predictive models that include sex and *APOE*. Early prediction of dementia will likely require integrating demographic and environmental information in addition to genetics.

Our comparisons also demonstrate the benefit of diversifying genomic studies of AD, adding to the growing calls for diversity across genetic research. The PRS‐CS model using summary statistics from cross‐population GWAS of AD consistently performed better and was more strongly associated with incident dementia than the PRS‐CS model using summary statistics from the European‐ancestry GWAS in admixed Black and Hispanic populations, despite the smaller sample size of the cross‐population GWAS. This finding is consistent with previous work examining other non‐dementia traits that demonstrated the superior performance of PRS derived from multi‐ancestry GWAS meta‐analyses compared to single‐ancestry GWAS (Gunn et al. 2025). Of note, neither of the PRS‐CS models significantly outperformed the restrictive C+T model with 15 SNPs derived from the IGAP GWAS representing European ancestry in the overall sample or Black and White subgroups. It's likely that, despite the smaller number of SNPs, this restricted set is more likely to tag regions that have true biological impact on risk of disease development, but more diverse studies are needed to capture population‐specific genetic architecture in the Chinese and Hispanic populations.

PRS are least predictive in individuals with high amounts of genetic admixture (i.e. those in the intermediate NFE‐like proportion group). We observed that PRS‐CSx performed best in the group with intermediate proportions of NFE‐like ancestry. This aligns with previous findings that have observed increased predictive performance in admixed groups when using a linear combination of summary statistics that combine ancestry‐specific effect sizes (Bitarello and Mathieson 2020). Nevertheless, the PRS‐CSx performance in this group remained lower than the best‐performing models in the high NFE‐like group, and the PRS derived from PRS‐CSx was not associated with incident dementia in this group (Supporting Figure 3). The limited association could be due to the relatively small sample size of the African Genome Resources Panel GWAS and the lack of GWAS information from studies with substantial AMR ancestry.

Our study has several limitations, most notably the reliance on ICD codes for dementia phenotyping in our primary analysis. Incident dementia cases were ascertained as “possible dementia” from hospitalization and death records using ICD codes, and this method likely underestimates the true incidence because a portion of individuals living with dementia will either not be hospitalized or have an alternative listed cause of death. However, the external validation of electronic health records by physician review and the association of both *APOE* genotype and our PRS with incident dementia suggest that dementia cases are true positives (Fujiyoshi et al. 2017). ICD‐based possible dementia classification is also not specific for AD. Because our PRS were trained using GWAS of clinically adjudicated AD while the target phenotype in MESA reflects all‐cause dementia, phenotype mismatch could be a cause of attenuated associations and reduced predictive performance. The all‐cause dementia phenotype may also help explain the relatively low *APOE* ε4 frequency among dementia cases, as non‐AD dementias are expected to have a weaker relationship with *APOE* ε4 than AD. We conducted sensitivity analyses to assess whether the findings were robust when using alternative case definitions. Specifically, we conducted analyses including clinically adjudicated probable dementia cases and additionally restricted cases to those with AD‐specific ICD codes in a separate analysis. Results were broadly consistent with the primary analysis, suggesting that the main conclusions were not driven solely by inclusion of non‐AD dementia. However, the number of adjudicated probable dementia cases was limited, and estimates in the AD‐specific ICD analysis were less precise because of the smaller number of events. Future studies of pathologically confirmed AD in diverse populations are needed to better evaluate the performance of AD‐specific PRS and to clarify whether the patterns observed here differ across dementia subtypes. Furthermore, there are larger GWAS of dementia‐by‐proxy phenotypes conducted in European ancestry samples that provide a larger pool of SNPs considered to be significantly associated with parental history of dementia. Due to the sub‐optimal dementia adjudication of our target data, we chose to prioritize depth of phenotyping over sample size. We also limited our summary statistics to variants identified in GWAS and, therefore, did not consider rare variants that may have large effects on AD. We were also limited to using GWAS from European and African ancestry populations due to the lack of large‐scale GWAS data from Hispanic/Latino and Chinese populations. PRS performance in these populations will likely improve as more diverse studies are conducted. In addition, novel polygenic risk approaches are constantly being developed and we are unable to test all possible methods. Instead, we selected methods that are shown to perform well in the absence of individual‐level validation data due to the lack of datasets that match the diversity of our target sample and are not included in the GWAS from which the summary statistics are derived. Finally, while Chinese participants were included in our study, the sample size was too small to detect differences in performance across PRS models and none of the models resulted in PRS associated with dementia in this subgroup.

While our findings show that current PRS models have modest predictive value, future AD GWAS in diverse populations have the potential to enhance their predictive power. Furthermore, the utility of PRS may extend beyond estimating the overall likelihood of disease development. Future research should focus on how PRS can be used to identify differences in disease pathogenesis and clinical trajectories using etiology‐confirmed cases of dementia and specifically AD in diverse populations. Incorporation of rare variants and leveraging functional annotation to develop pathway‐specific scores will further enhance the precision and translational value of PRS. For now, it seems there is no tradeoff between simplicity and accuracy; a simple C+T approach with only the most highly significant SNPs offers comparable prediction of dementia in populations with diverse ancestry.

## Ethics Statement

MESA activities include central IRB and local IRB oversight. Informed consent was obtained from each participant at baseline and updated at each examination. Approval was received at each site from the local institutional review board for each examination.

## Conflicts of Interest

The authors declare no conflicts of interest.

## Supporting information

Supporting File

## Data Availability

The data that support the findings of this study are available from the Multi‐Ethnic Study of Atherosclerosis. Restrictions apply to the availability of these data, which were used under license for this study. Data are available from the author(s) with the permission of the Multi‐Ethnic Study of Atherosclerosis. Data underlying this article will be available upon request and with appropriate approvals.
