# Supplementary material for: Polygenic Risk Scores for Incident Dementia in the Multi‐Ethnic Study of Atherosclerosis
Source: Genet Epidemiol. 2026 Jun 23;50(5):e70046. doi: 10.1002/gepi.70046 (PMC13288446; doi:10.1002/gepi.70046)
Supplement: Supplementary file 1 — Supporting File [file GEPI-50-0-s001.docx]

### **Supplementary Figure 1. Global Ancestry Proportions for MESA participants.**

This plot represents the proportion of African (AFR), Amerindian (AMR), East Asian (EAS) and Non-Finnish European (NFE) for each participant, separated by self-reported race/ethnicity. Each participant represents one column. Reference samples are from gnomAD v3.1.


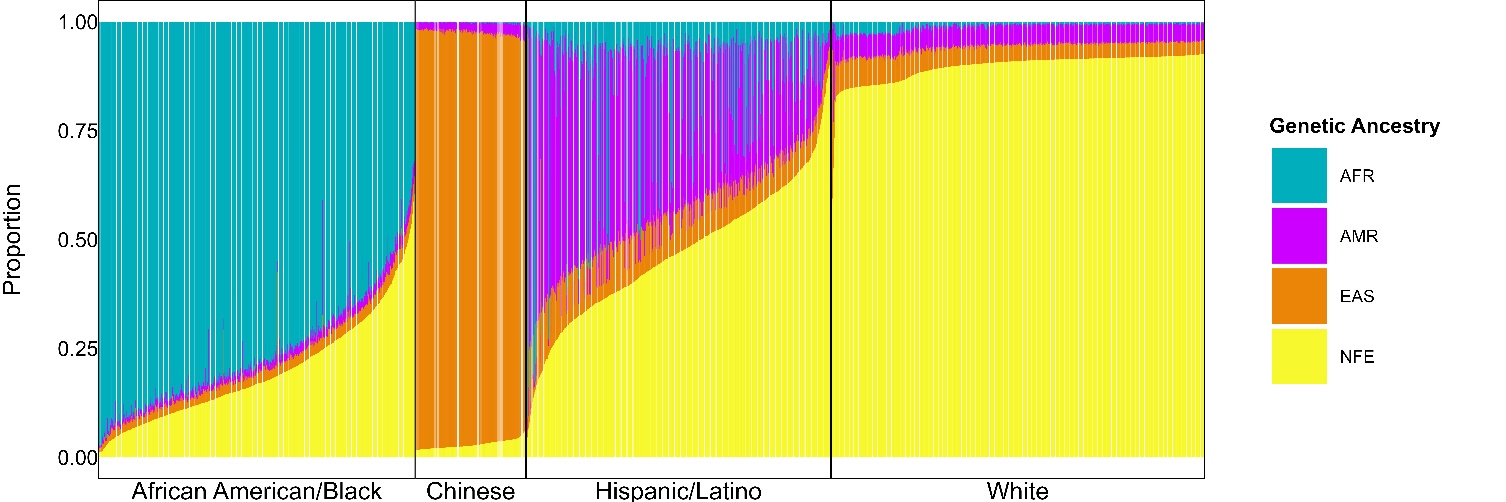


### **Supplementary Figure 2. Polygenic risk score distributions by self-reported race/ethnicity, pre- and post- calibration by principal components.**

**Pre-Calibration**


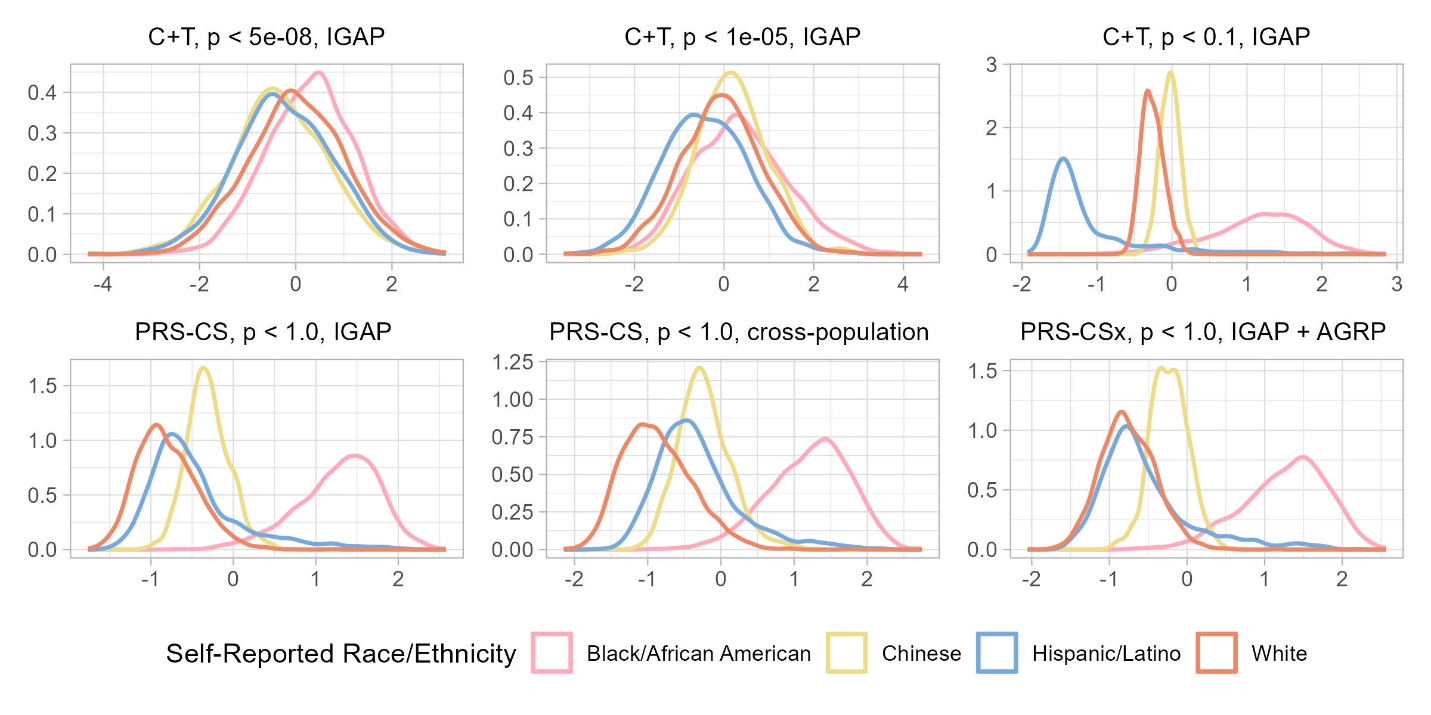

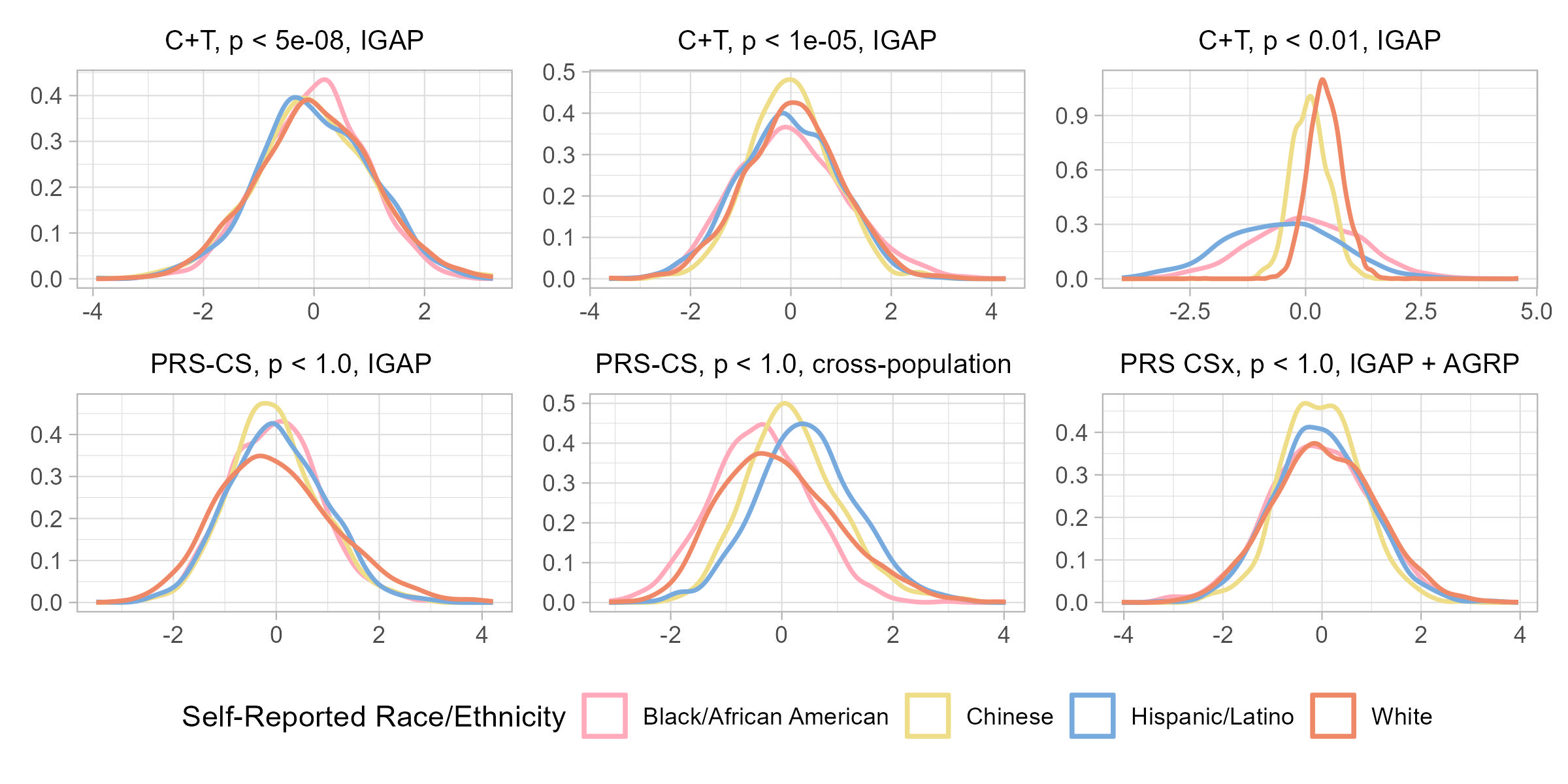


**Calibrated**

**Supplementary Figure 3.** **Association between adjusted PRS and incident dementia stratified by proportion of NFE ancestry.**

*NFE = proportion of ancestry similar to 1000 Genomes non-Finnish European references.


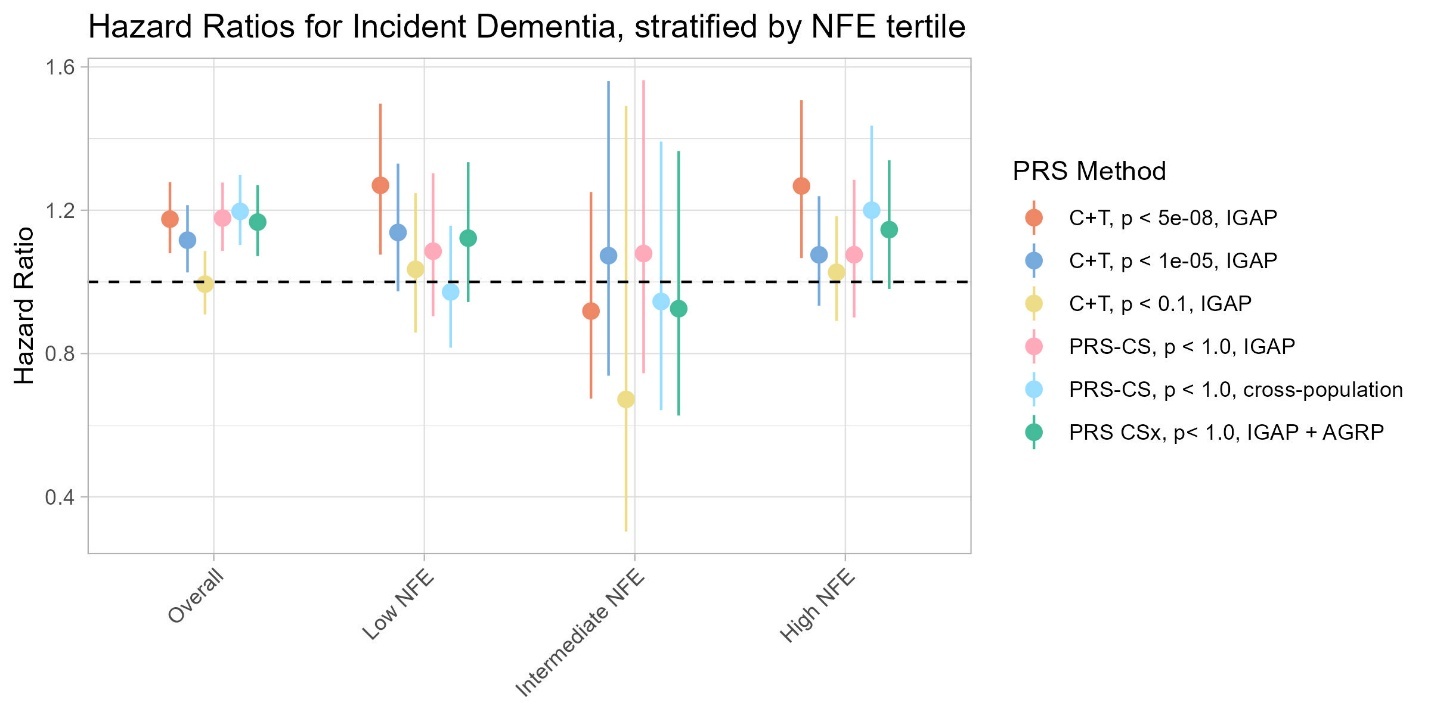


**Supplementary Figure 4.** **Association between adjusted PRS and incident AD (based on ICD codes)**


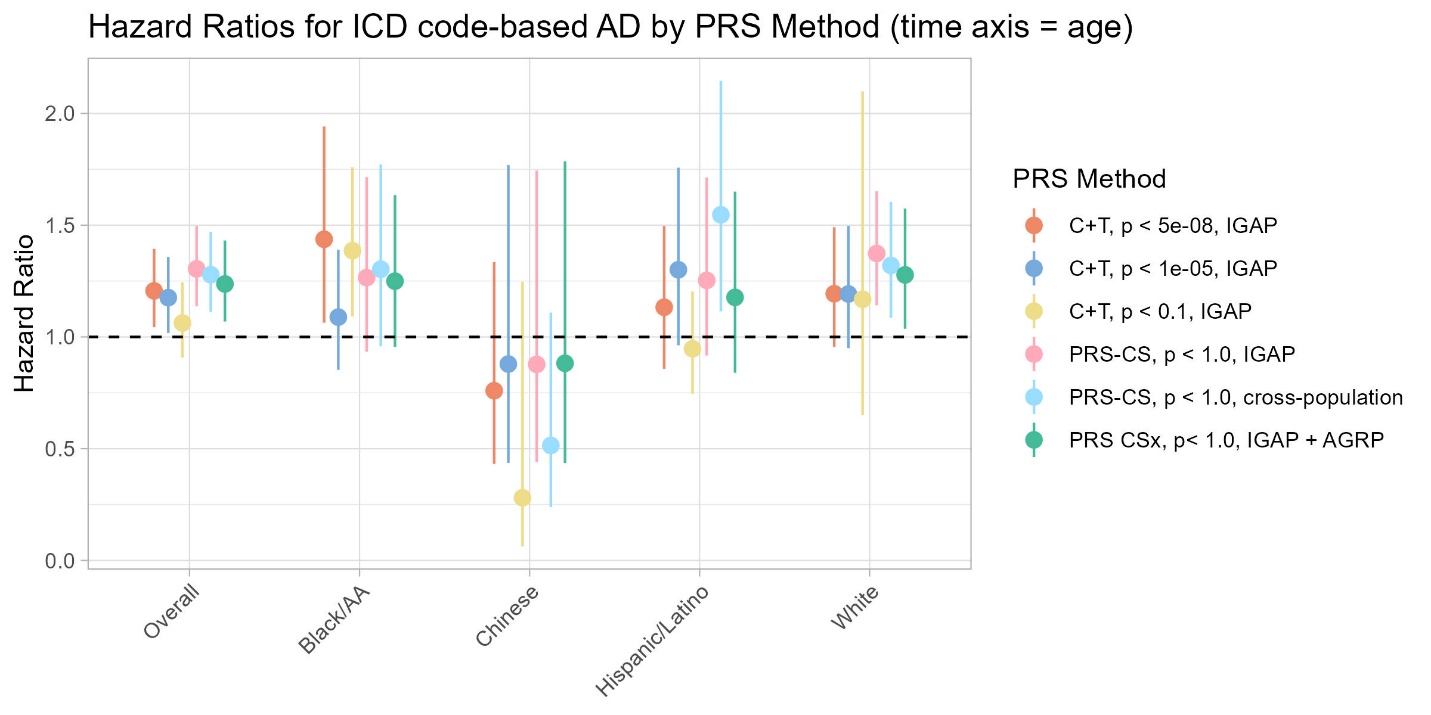


### **Supplementary Figure 5. Association between adjusted PRS and dementia case-control status**


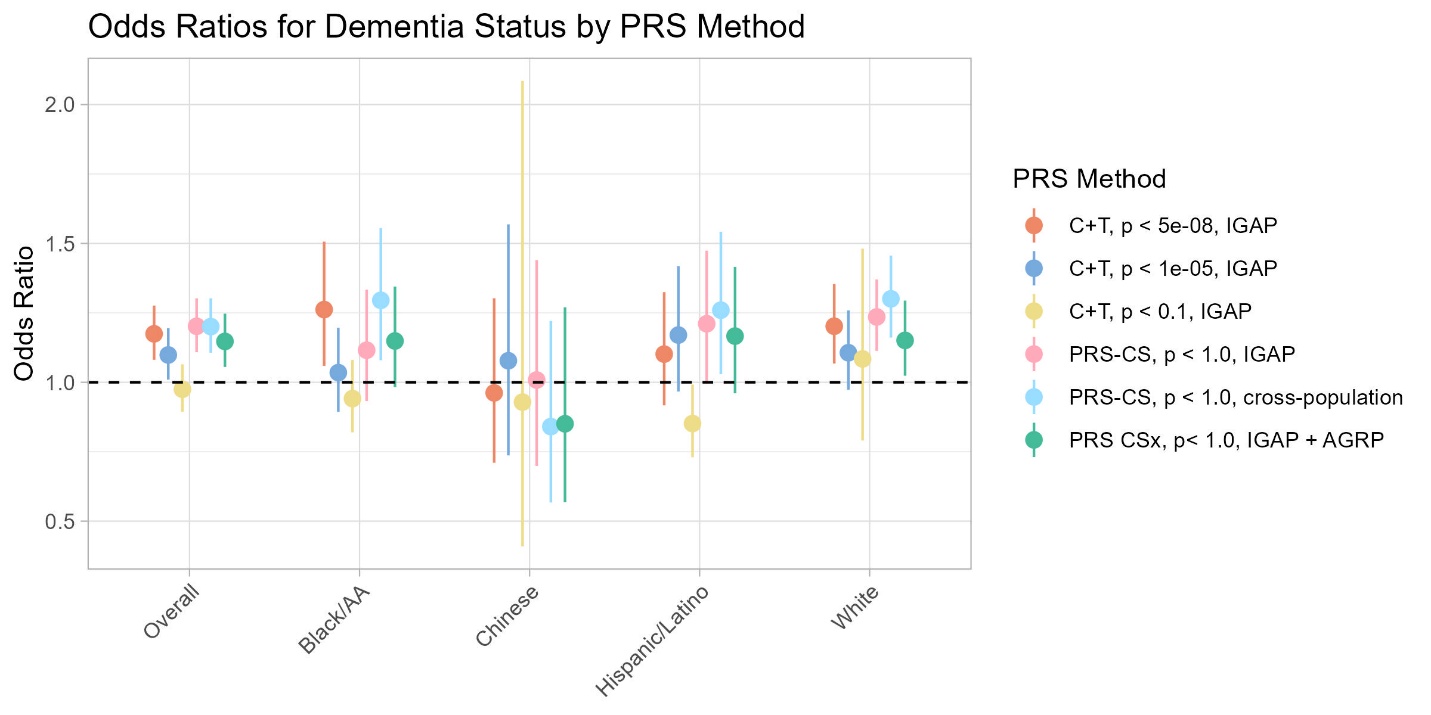

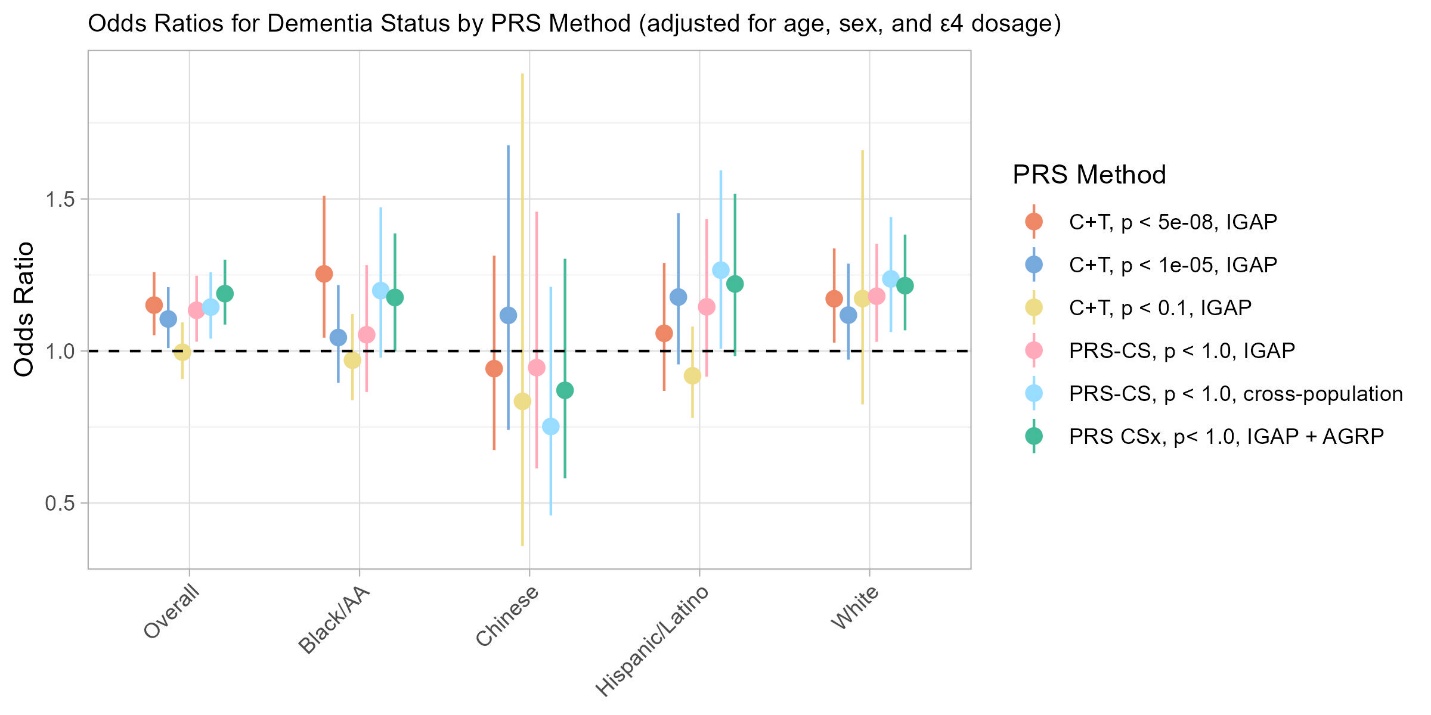


### **Supplementary Figure 6. PRS predictive performance measured by AUC.**

The left-hand panel represents the predictive performance of a univariate model where the PRS alone are used to predict dementia status. The right-hand panel represents the predictive performance of a model that includes the PRS alongside age, sex, and *APOE* *ε4* carrier status.


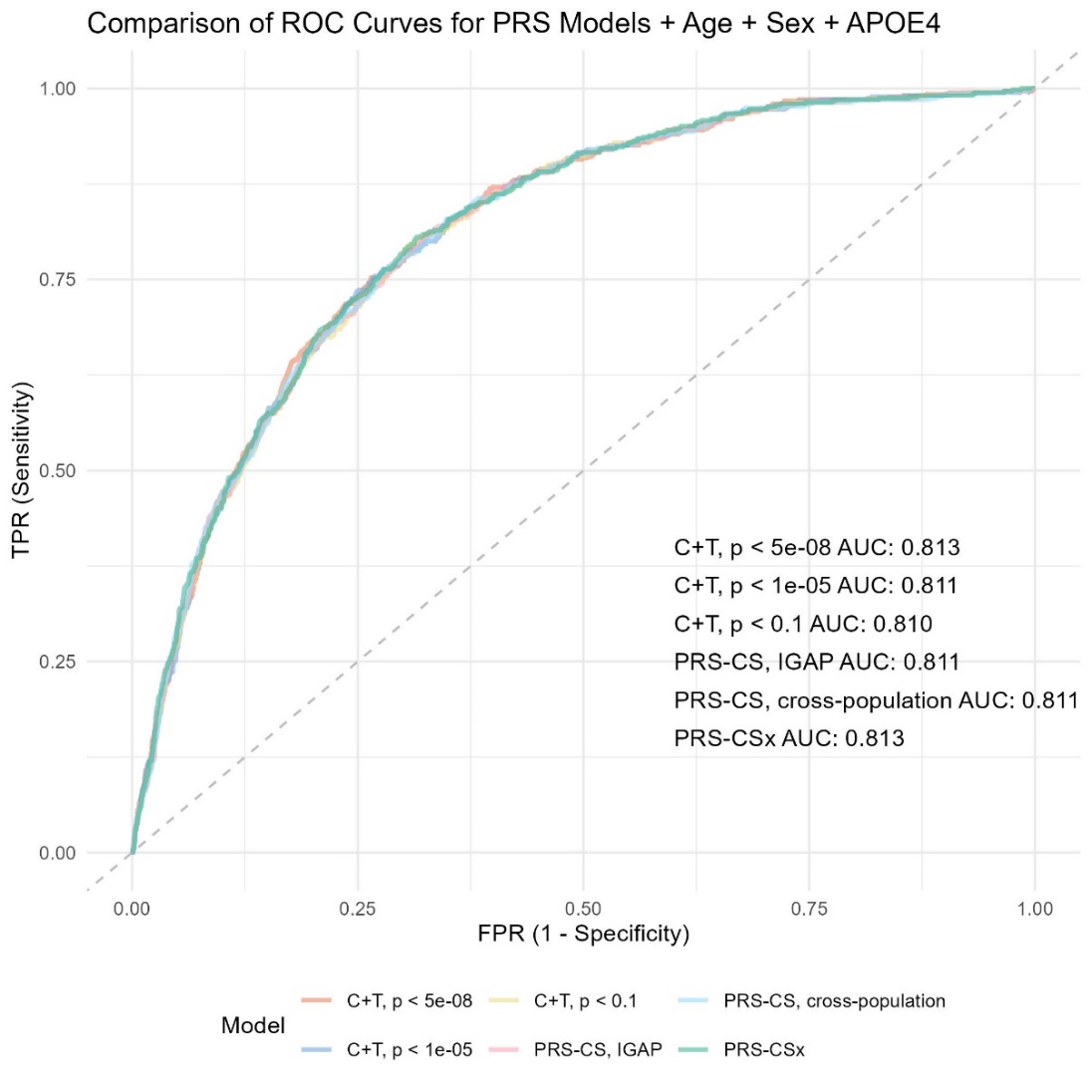

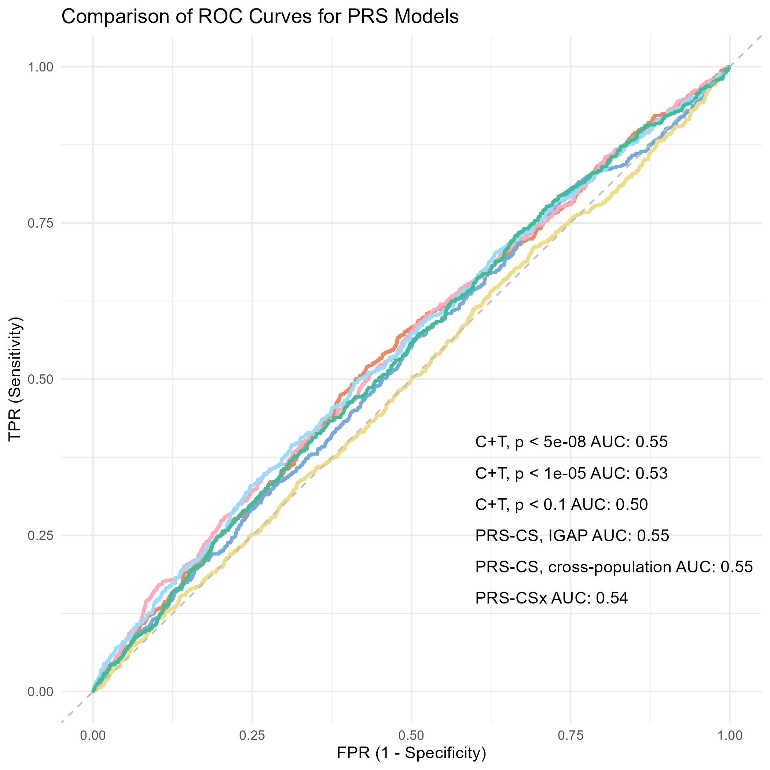

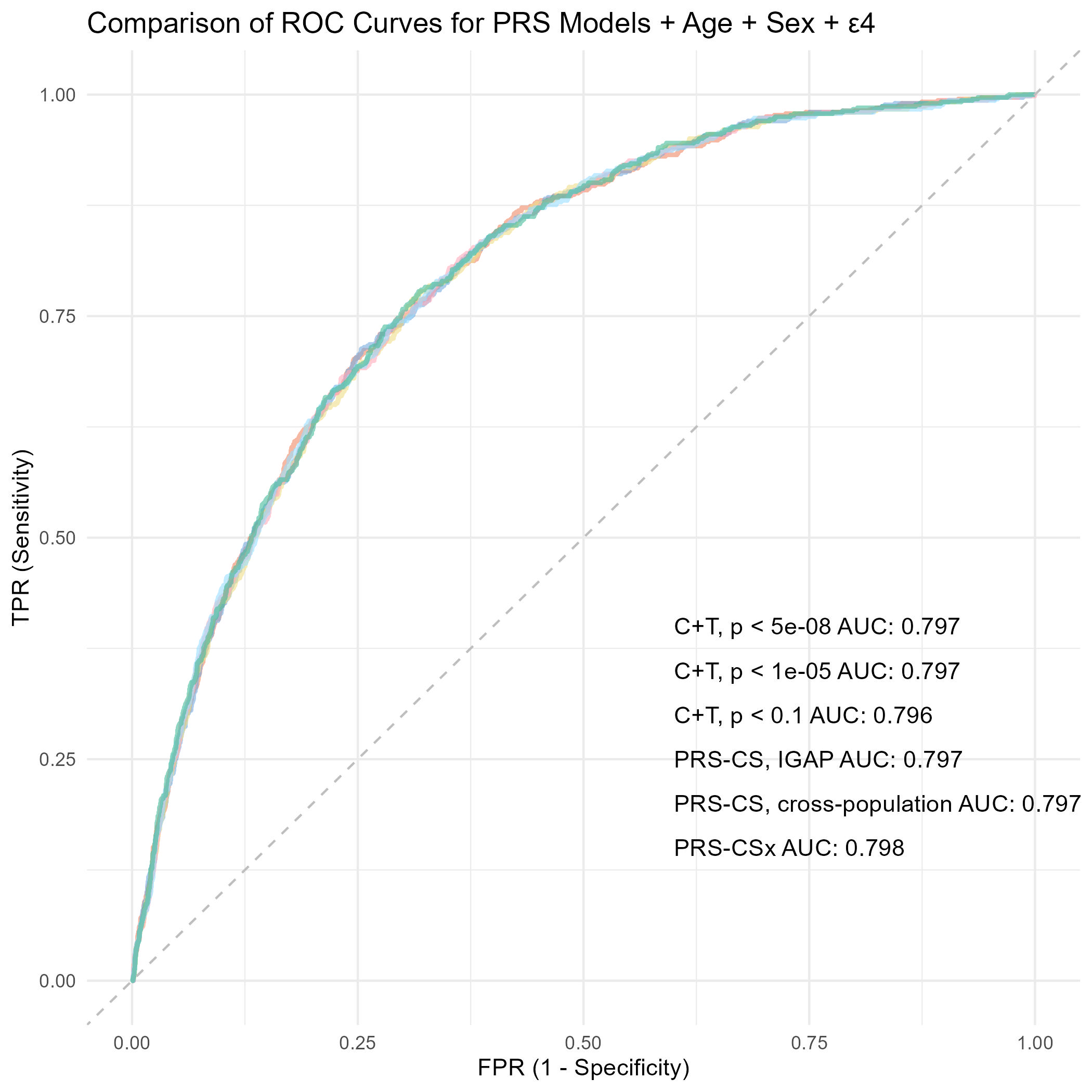


### **Supplementary Figure 7. PRS predictive performance comparisons stratified by proportion of NFE-like ancestry**

Comparisons of PRS predictive performance as measured by Harrell’s C across tertiles of NFE-like ancestry.

*NFE = proportion of ancestry similar to 1000 Genomes non-Finnish European references.


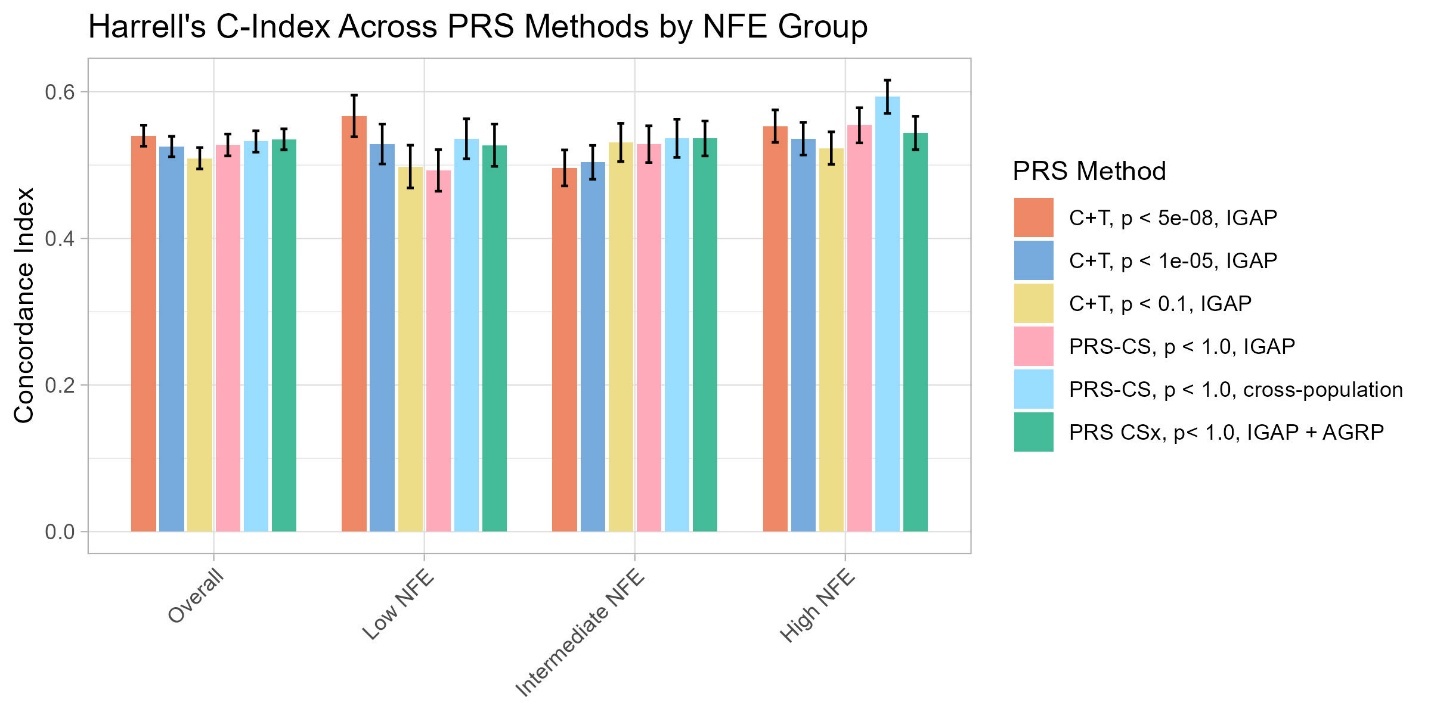


### **Supplementary Figure 8. PRS predictive performance comparisons for incident AD (based on ICD codes)**


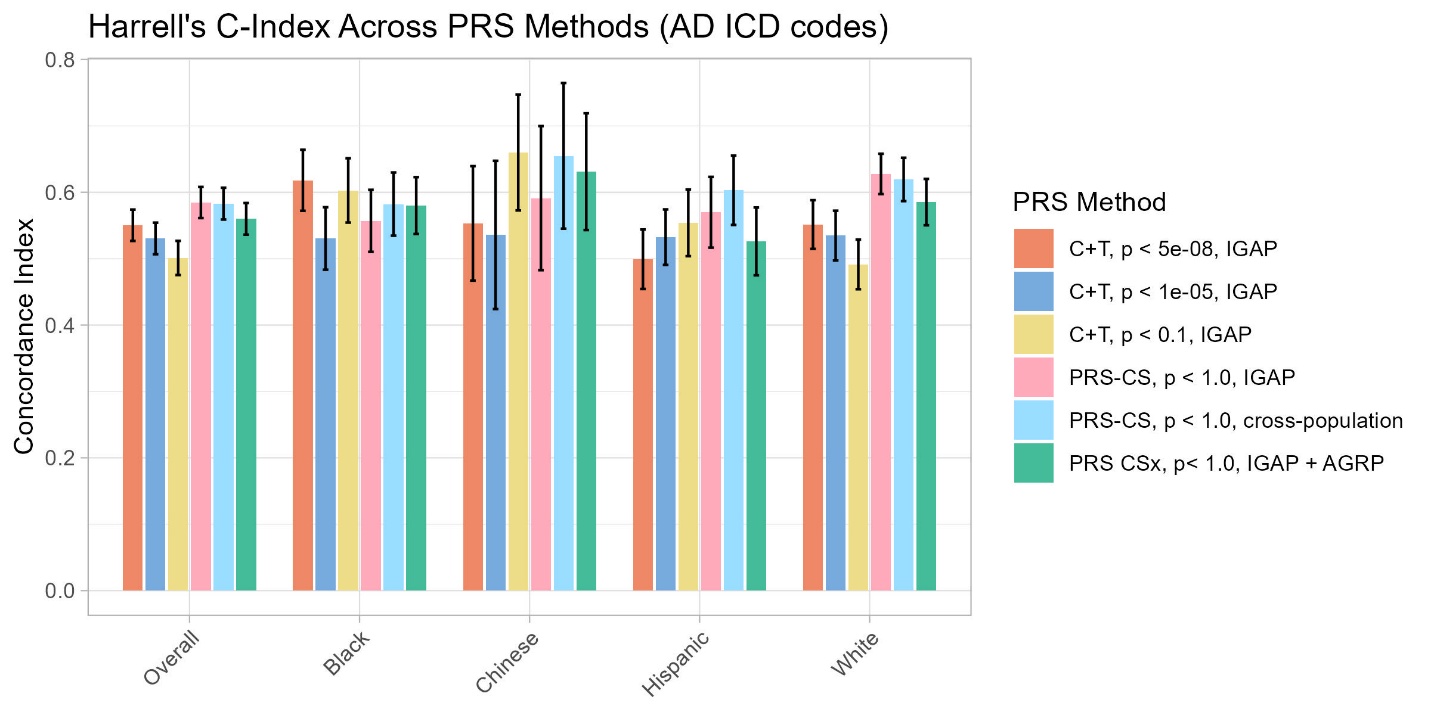


### **Supplementary Table 1. Possible and probable dementia case proportions**

|  | N | % of total |
| --- | --- | --- |
| MESA cohort followed through 2018 | 6338 | 100 |
| Possible dementia by ICD code (through 2018) | 560 | 8.8 |
| MESA MIND cognitive adjudication | 2329 | 36.8 |
| Probable dementia by cognitive adjudication | 71 | 1.1 |

### **Supplementary Table 2. Mean age at baseline by self-reported race/ethnicity**

| **Self-reported race/ethnicity** | **Mean Baseline Age (Std. Dev.)** |
| --- | --- |
| Black | 62.3 (10.1) |
| Chinese | 62.4 (10.4) |
| Hispanic | 61.3 (10.3) |
| White | 62.6 (10.3) |

### **Supplementary Table 3. *APOE* ɛ4 prevalence by baseline age category**

|  | **N** | **% ε4 carriers** | **% ε4/ε4** |
| --- | --- | --- | --- |
| **45-54** | 1769 | 28.5 | 2.7 |
| **55-64** | 1703 | 27.3 | 2.3 |
| **65-74** | 1860 | 25.2 | 2.4 |
| **75-84** | 893 | 25.3 | 1.8 |
